# Prevalence and Risk Factors of Dry Eye Syndrome Among Medical Students in the Northern Philippines: A Cross-Sectional Survey

**DOI:** 10.7759/cureus.86740

**Published:** 2025-06-25

**Authors:** Mary Arsenia N Mondiguing, Karl Coycoyen, Monique Goygoyan, Jay Adrian Taguiling, Kc Chakiwag, Winston Calde, Kiarei Dao-ayan, Clar Renzel Bombase, Mark Rigor, Athena May Catores

**Affiliations:** 1 Ophthalmology, Saint Louis University School of Medicine, Baguio City, PHL

**Keywords:** dry eye syndrome, medical school students, ocular surface disease index (osdi), perceived stress scale-4 (pss-4), visual display terminals

## Abstract

Introduction

Dry eye syndrome (DES) accounts for the majority of ophthalmological consultations and should be considered a high-priority health concern. DES can be progressive and has significant consequences for an individual's vision and quality of life. This study aims to fill the information gap regarding the prevalence and risk factors of DES among medical students at Saint Louis University (SLU) in the northern part of the Philippines during the "New Normal" period.

Methods

A cross-sectional survey was conducted among 236 medical students. The dependent variable, the Ocular Surface Disease Index (OSDI), and independent variables were assessed using a questionnaire created with Google Forms (Google, Inc., Mountain View, CA). The Perceived Stress Scale-4 (PSS-4) was used to determine the level of stress of the students. Descriptive and analytical statistics were used to determine the risk factors associated with the OSDI scores of the participants.

Results

The prevalence of DES, as determined by the OSDI questionnaire, was 194 out of 236 (82.20%). The mean OSDI score of the cohort was 30.16 ± 6.99, with a 95% confidence level. This study revealed that the factors of sex, error of refraction (EOR), and psychological stress showed significant correlations with the OSDI score, as determined by Chi-square analysis with p-values of 0.013, 0.001, and 0.006, respectively. The female sex and those with errors of refraction (EOR) had a higher risk of developing DES. There is also a direct relationship between psychological stress and DES. The higher the perceived psychological stress, the higher the OSDI score. Psychological stress among the students was at 231/236 (97.87%).

Conclusion

Dry eye syndrome is increasingly recognized as a significant health issue among young individuals due to the increasingly demanding nature of their lifestyles. This study identified a high prevalence of dry eye syndrome among medical students with female sex, error of refraction, and a high level of perceived stress as the significant risk factors. A longitudinal study involving different medical schools in the country, including clinical tests for DES, is recommended to better elucidate the relationship among the various risk factors. The results of this study serve as a basis for crafting policies to increase awareness, modify risk factors, and implement appropriate preventive measures.

## Introduction

Dry eye syndrome (DES) accounts for the majority of ophthalmological consultations and should be considered a high-priority health concern. DES can be progressive and have significant consequences for an individual's vision and quality of life. People of all ages frequently experience dry eye syndrome, and this condition is often a chronic issue that remains underappreciated, misdiagnosed, and undertreated. DES is a multifactorial ocular surface and tear disorder that leads to various conditions, including visual disturbances, symptoms of discomfort, decreased tear production, increased evaporation, and hyperosmolarity of the tear film [1,2]. Common complaints associated with dry eye syndrome include redness, excessive tearing, itchiness, burning, irritation, photophobia, dryness, foreign body sensation, mucoid eye discharge, and visual disturbances. Evidence suggests a variety of intrinsic and extrinsic risk factors that predispose individuals to develop DES, including gender, age, medical and ocular conditions, comorbidities, medications, social and environmental factors, and the individual's perceived stress [3].

Literature suggests that DES affects both men and women of all ages; however, recent studies showed a significant increase in the prevalence of DES among women and young individuals [3]. Ocular conditions associated with DES include errors of refraction (EOR), use of corrective lenses, refractive and cataract surgeries, eye diseases, and eye injuries. Certain medications and comorbidities may also be associated with the development of ocular diseases. Social and environmental factors may include smoking, staying indoors in air-conditioned rooms, duration and frequency of use of visual display terminals (VDTs), sleep deprivation, prolonged use of face masks, and perceived stress [3,4]. Sleep deprivation has been proven to cause and exacerbate DES more often than the use of computers, television, or video, and leisure reading [4,5]. Several studies suggest that the rise in ocular discomfort during the pandemic is largely attributed to dry eye syndrome, primarily due to the prolonged use of face masks, resulting in mask-associated dry eye [6]. Current studies suggest that the COVID-19 pandemic has exacerbated these risk factors, leading to a marked increase in the occurrence of DES cases [7].

Since the COVID-19 pandemic, extending to the "New Normal," there has been a tremendous transition in daily life and activities, including the adoption of remote learning and work modalities [8-13]. The transition to the "New Normal" introduced various challenges that extended beyond health and safety concerns. Medical students were not exempt from this and were, in fact, among the high-risk groups for DES [14]. The increased screen time and prolonged use of digital devices for educational purposes, coupled with sleep deprivation, prolonged use of face masks, and psychological stressors such as economic and health crises, notably raised concerns about the potential exacerbation of dry eye syndrome among medical students globally. Adapting to this new educational situation has meant not only adjusting to novel learning environments but also facing an array of new health-related issues, including DES. A search of published articles on DES among medical students showed studies in India [4], Poland [9], Brazil [15], Serbia [16], and Dubai [17], but none in the Philippines. Further, as of the writing of this paper, there were no published articles on DES during the "New Normal." This study aims to fill the information gap regarding the prevalence of DES among medical students at Saint Louis University (SLU) in the northern part of the Philippines during the "New Normal" period. In particular, we aim to determine the association between DES and the following factors: age, sex, error of refraction, prolonged use of contact lenses, cumulative use of video display terminals (VDTs), smoker or exposure to smokers, average hours in air-conditioned rooms, regular medication intake, allergies, presence of comorbidities, prolonged use of face masks, sleeping hours, and perceived stress; it aimed to ascertain the risk factors associated with DES among the medical students.

## Materials and methods

Study design

This study employed a cross-sectional survey design. The dependent variable, the prevalence of dry eye syndrome, and independent variables were assessed using a questionnaire created with Google Forms (Google, Inc., Mountain View, CA). Descriptive and analytical statistics were used to determine the risk factors associated with the OSDI scores of the participants.

Study population

The study population consisted of first- to fourth-year medical students currently enrolled at Saint Louis University in Baguio City for the academic year 2023-2024. The study was conducted from September 25, 2023, to March 30, 2024. A total of 609 students were enrolled in the Doctor of Medicine degree program. A sample size of 236 was calculated using OpenEpi Info version 7.2 (Dean AG, Sullivan KM, Soe MM. OpenEpi: Open Source Epidemiologic Statistics for Public Health, Version. www.OpenEpi.com, updated 2013/04/06), with a 95% confidence interval (CI) and a 5% margin of error. A convenience sampling procedure was used for recruitment. The participants included in the study were the medical students who had completed the survey. Those with ocular conditions that predispose to DES, such as a history of refractive surgery, recent ocular trauma or surgery, and ocular infections, were excluded. The investigators managed to obtain completely answered questionnaires from 236 medical students or 100% of the computed sample size.

Data collection

Since the research promoter is a faculty member of the SLU School of Medicine, the student co-investigators presented a brief background and the objectives of the study to each year level of the medical students. The Ocular Surface Disease Index (OSDI) [18] and the Perceived Stress Scale-4 (PSS-4) [19] questionnaires were also introduced. Thereafter, the letter of invitation, informed consent form (ICF), and questionnaires were emailed to all medical students from first to fourth years. Responses were tallied as they arrived. A second round of verbal invitations was conducted per class when the sample population was insufficient. Complete responses were included until completion of the target size. Fortunately, there was a well-representation of all year levels.

The informed consent form (ICF) and questionnaire, which included participants' demographics, related risk factors, and validated questionnaires on OSDI and PSS-4, were emailed to all participants using their university email addresses. The responses were divided into five sections: demographic profile (age, sex, and year level), medical and ocular history (eye disease, refractive surgery, eye injuries, error of refractions, duration of contact lens wear, presence of comorbidities, allergies, and regular medication intake), social and environmental history (smoker or exposure to smokers, average hours indoors in air-conditioned rooms in hours/day, duration of sleeping time, use of face masks in hours/day, and cumulative use of video display terminals (VDT) in hours/day such as projectors, laptop, desktop, computers, and smart gadgets), the validated dry eye symptom questionnaire (Ocular Surface Disease Index (OSDI)), and the validated questionnaire on perceived stress (Perceived Stress Scale-4 (PSS-4)).

Assessment of Dry Eye Syndrome

The Ocular Surface Disease Index (OSDI) was used to evaluate DES. This instrument is a questionnaire composed of 12 items that evaluate dry eye symptoms experienced within the past week, prior to answering the questionnaire. It is a validated questionnaire used to determine the severity of dry eye syndrome, ranging from normal to mild, moderate, and severe. The OSDI examined three sections: subjective ocular symptoms, limitations in performing daily activities due to eye problems, and influence of environmental conditions on eye discomfort. Responses to each question ranged from 0 to 4, where 0 represents none of the time; 1, some of the time; 2, half of the time; 3, most of the time; and 4, all of the time. The collected final scores ranged from 0 to 100, with higher scores representing more significant disability: normal (0-12), mild (13-22), moderate (23-32), and severe (33-100) [4]. DES is present for an OSDI score of 13 and above [4].

Assessment of Perceived Stress

The psychological stress of the students was determined using the Perceived Stress Scale-4 (PSS-4) questionnaire. The PSS-4 is a validated instrument that measures an individual's psychological stress level and the degree to which situations in one's life are appraised as stressful. The Perceived Stress Scale has been modified from a 14-item scale to a 10-item scale and eventually to a four-item scale version, which is easier to access, complete, and assess. The internal consistency of PSS-4 was determined to be sufficient, with Cronbach's α of 0.82, for a four-item scale. It is utilized in several studies to assess the stress perception of different populations [19,20].

The Perceived Stress Scale-4 (PSS-4) asks questions about the participant's feelings and thoughts during the last month. The participants will indicate how often they have felt or thought a certain way. Answers are rated as 0 = never, 1 = almost never, 2 = sometimes, 3 = fairly often, and 4 = often. Questions 1 and 4 are graded as 0 = never, 1 = almost never, 2 = sometimes, 3 = fairly often, and 4 = very often, whereas questions 2 and 3 are graded as 4 = never, 3 = almost never, 2 = sometimes, 1 = fairly often, and 0 = very often. The scores range from 0 to 16. Higher scores correlated with more stress [19].

Data management

The questionnaires were distributed using Google Forms for easier database organization and analysis. The student investigators conducted the questionnaires in between classes. Data obtained from Google Forms was automatically imported into Microsoft Excel (Microsoft Corp., Redmond, WA) and cross-referenced with the summary analytics from the Google Forms survey questionnaires. Given the summary analytics, data that met the exclusion criteria and had missing values were excluded from the analysis. Each data point was assigned a unique numerical code to facilitate accurate and efficient analysis. The numerical codes were assigned manually and randomly to avoid bias in coding, ensuring that each piece of data was secure and kept confidential. SPSS version 21 (IBM Corp., Armonk, NY) was used for data analysis.

Statistical analysis

The dependent variable was the prevalence of dry eye syndrome (DES). Independent variables include age, sex, error of refraction (myopia, hyperopia, and astigmatism), prolonged use of contact lenses, cumulative use of video display terminals (VDT) in hours/day, smoker or exposure to smokers, average hours in an air-conditioned room, regular medication intake, allergies, presence of comorbidities, prolonged use of face masks, sleeping hours, and perceived stress. Data were interpreted using descriptive statistics, which included frequency, percentage, mean, and standard deviation (SD). The Chi-square test, Spearman correlation coefficient, and least significant difference (LSD) were used to summarize the relationship between OSDI and the different variables. The direction will always be positive or negative. Analysis of variance (ANOVA) was used to quantify the strength of association among the categorical variables. A level of p<0.05 was deemed statistically significant.

Ethical considerations

This study was conducted in accordance with the ethical principles outlined in the Declaration of Helsinki and the National Ethical Guidelines for Research Involving Human Participants (NEGRIHP). The University Research and Innovation Center approved the scientific and ethical soundness of the protocol.

Autonomy, in the form of voluntary participation by respondents and the provision of informed consent, was observed. The study included medical students who voluntarily signed consent forms and completed the survey. Equitable selection was ensured, considering the potential benefits and risks to different groups. The participants were not required to write their names on the questionnaires to ensure anonymity. All clinical data were kept confidential and anonymous. The confidentiality of results is governed by the Data Privacy Act of the Philippines, also known as Republic Act No. 10173. Only the primary investigator and three of the co-investigators had access to the data. Data will be stored for three years and then discarded. The investigators declared no conflicts of interest that could arise from financial, familial, or proprietary considerations. No expenses were incurred, and no compensation was offered to the participants.

## Results

Prevalence of dry eye syndrome

**Figure 1:**
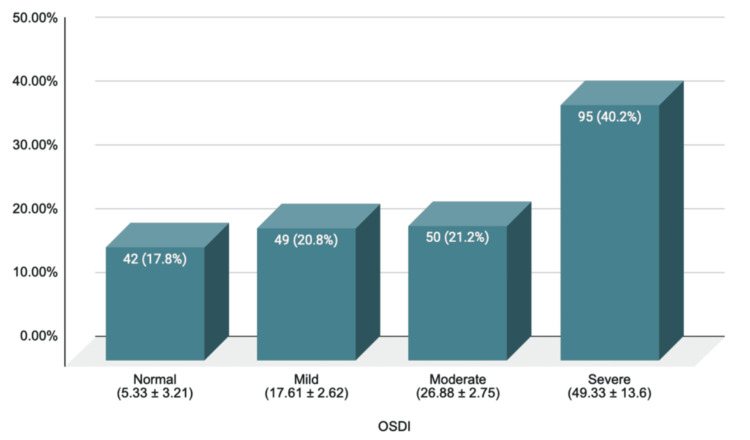
Prevalence of dry eye syndrome among study participants using the OSDI questionnaire (N = 236) OSDI: Ocular Surface Disease Index

The prevalence of dry eye syndrome is shown in Figure 1. The mean OSDI value of the cohort was 30.16 ± 6.99, with a 95% confidence level. The mean value is higher than the cutoff value of 12. Based on the OSDI questionnaire alone, the prevalence of DES in the study was 82.20%.

Sociodemographic, clinical, social, and environmental profile

The sociodemographic, clinical, social, and environmental profile is shown in Table 1. A total of 236 medical students representing 100% of the computed sample size participated in the study. The age range was 20-30 years, with a female preponderance of 170 (72.03%). Male participants had moderate DES compared to severe DES among female participants. A total of 196 (83.05%) participants had errors of refraction, and 102 (43.22%) had been using contact lenses. There is a strong correlation (p < 0.001) between the severity of ocular surface disease and error of refraction, but no association (p = 0.091) with the use of contact lenses.

**Table 1:** Baseline sociodemographic, medical, ocular, social, and environmental profile with OSDI scores of the study cohort (N = 236) OSDI: Ocular Surface Disease Index, SD: standard deviation, DES: dry eye syndrome, VDTs: video display terminals, PSS-4: Perceived Stress Scale-4, DM: diabetes mellitus

Demographics	Frequency		OSDI score	
	Number	%	Mean	SD
Age				
20-25	178	75.42	30	18
26-30	58	24.58	30	21
Sex				
Male	66	27.97	24	16
Female	170	72.03	33	20
Year level				
1st year	64	27.12	29	18
2nd year	47	19.91	32	20
3rd year	61	25.85	33	19
4th year	64	27.12	27	19
Error of refraction				
Myopia	63	26.69	32	19
Hyperopia	2	00.85	15	15
Astigmatism	131	55.51	33	19
None	40	16.95	17	14
Contact lens wear				
None	134	56.78	28	20
≤1 year	38	16.10	32	17
2-3 years	12	5.08	38	17
>3 years	52	22.03	34	19
Comorbidities				
None	222	94.07	30	19
Hypertension	13	5.51	29	19
DM	1	0.42	29	
Allergies				
Yes	90	38.14	34	19
No	146	61.86	28	19
Regular medication intake				
None	207	87.71	29	19
Contraceptives	11	4.66	40	22
Analgesics	10	4.24	37	19
Antihypertensive	7	2.97	30	15
Anti-hyperglycemic	1	0.42	29	
Cumulative use of video display terminals				
<4 hours	5	2.12	22	19
4-8 hours	68	28.81	26	19
>8 hours	163	69.07	32	19
Smokers/exposed to smokers				
Yes	40	16.95	29	20
No	196	83.05	30	19
Average hours in air-conditioned room/day (hours)				
<4 hours	109	46.18	29	20
4-8 hours	85	36.02	29	17
>8 hours	42	17.80	36	22
Sleeping hours				
<4 hours	46	19.49	33	18
4-8 hours	185	78.39	30	20
>8 hours	5	2.12	22	16
Use of face masks (hours/day)				
<4 hours	121	51.27	29	17
4-8 hours	61	25.85	32	20
>8 hours	54	22.88	29	22
PSS-4				
0-4	5	2.12	27	17
5-8	124	52.54	26	18
9-12	103	43.64	36	20
13-16	4	1.69	15	22

Considering that the study group belongs to the young population, only 14 (5.93%) of them had comorbidities, and 90 (38.14%) had allergies. Only 29 (12.29%) were on maintenance medications, which include antihypertensives, hypoglycemics, anti-allergy medications, contraceptives, and/or hormonal treatment.

Psychological stress was tested using the Perceived Stress Scale-4 (PSS-4). Higher scores of 6 and above were correlated with more stress. Out of 236 students, 231 (97.88%) had scores of 5 and above.

Association between dry eye syndrome and the various risk factors

The association of DES and the independent variables is shown in Table 2. This study revealed that the factors of sex, error of refraction, and psychological stress showed significant correlations with the OSDI score, as determined by Chi-square analysis with p-values of 0.013, 0.001, and 0.006, respectively. The female sex and those with errors of refraction (EOR) had a higher risk of developing DES. There is also a direct relationship between psychological stress and DES. The higher the perceived psychological stress, the higher the OSDI score. There is no relationship between sleeping time and DES (p = 0.196). However, Pearson's two-tailed correlation between PSS and sleeping time was significant at the 0.05 level (p = -0.141) and using Chi-square (p = 0.030). There is an inverse correlation where the shorter the sleeping time, the higher the psychological stress. The duration of continuous VDT use did not show a relationship with DES using the Chi-square test (p = 0.124). However, the analysis of variance for the use of VDT showed a significant difference among the different categories of VDT (p = 0.040). The least significant difference (LSD) was used to determine which among the categories of VDT is significantly different and revealed that the mean score on OSDI for >8 hours of use of VDT (mean: 32 ± 19) is significantly higher than the mean OSDI score in the 4-8 hours of use of VDT (mean: 26 ± 19), but comparable with the mean OSDI score for <4 hours (mean: 22 ± 19). All other comparisons showed no statistical difference. There was a highly significant association between psychological stress and duration of use of VDT, using the Chi-square test (p = 0.006). The remaining risk factors tested showed no significant correlation with OSDI scores.

**Table 2:** Association of OSDI score and the different risk factors *Significant difference (p < 0.05) using Chi-square test A correlation between DES and PSS-4 of p = 0.017 and p = 0.0001 using Pearson's and Spearman's correlation, respectively, was found to be significant. VDTs: video display terminals, PSS-4: Perceived Stress Scale-4, OSDI: Ocular Surface Disease Index, DES: dry eye syndrome

Risk factors	p-value
Demographic profile	
Sex	0.013*
Age	0.644
Year level	0.243
Medical/ocular risk factors	
Error of refraction	0.001*
Contact lens wear	0.091
Comorbidities	0.66
Medications	0.333
With allergies	0.054
Social and environmental factors	
Smoker/exposed to	0.732
Use of facial masks	0.23
Air-conditioned room	0.427
Sleeping hours	0.196
Use of VDTs	0.124
PSS-4	0.006*

## Discussion

Prevalence of dry eye syndrome (DES)

The mean OSDI score of the study cohort was 30.16 ± 19.2, which falls within the moderate range of DES. The prevalence of DES in this study was 82.2%, which is higher than the published prevalence among the same study groups from other countries based on the same OSDI criteria score of 13 and above. Cross-sectional surveys among medical students have shown a prevalence of 55.19% among Indians [4], 57.1% among Poles [10], 59.56% among Brazilians [15], 60.5% among Serbians [16], and 62.6% among Emiratis [17]. The close incidence among these groups can be due to similar risk factors, such as age group, prolonged use of VDTs, and higher stress levels among medical students [10]. The prevalence obtained in this study is also higher compared to the prevalence of DES during the COVID-19 pandemic, at 61.0% (95% CI: 51.8%-70.2%) globally and 56.7% (95% CI: 45.3%-68.1%) in Asia, based on a systematic review and meta-analysis of 11 articles from 2020 to 2022 [21]. A study among Indian medical students revealed a significant difference (p < 0.05) in DES cases between the pre-pandemic period (41.5%) and the pandemic period (55.19%) [4]. The higher prevalence obtained in this study can be attributed to the difference in the time setting, which occurred during the "New Normal," when students were exposed to greater psychological stress and longer use of VDTs. In retrospect, our study was also conducted in a city with an altitude of approximately 1,500 meters above sea level, where the weather is colder. High altitude, defined as a height of at least 2,400 meters above sea level, is characterized by low humidity and increased evaporation of tears. The moderate altitude of the study site could be another factor contributing to the higher prevalence; however, this may require further comparison among the same population in different areas or at varying altitudes within the country.

Association between DES and age and sex

Studies showed that older age and female sex have a higher risk of developing DES or ocular surface disease [4,10,21]. This study found a significant correlation between sex and OSDI scores (p = 0.013), which concurred with the findings of previous studies. The male-to-female ratio is 1:2.6, with mean OSDI scores of moderate (24 ± 16) and severe (33 ± 20) for male and female participants, respectively. Hormonal factors, including sex hormones, insulin and insulin-like growth factors, thyroid hormones, sex chromosome complement, and epigenetics, increase the vulnerability of women to DES [10,22]. In contrast, the medical students in this study revealed no significant correlation between age and OSDI score, presumably due to the close age range of the participants. Of the participants, 75% were 20-25 years old, and the rest were 26-30 years old.

Association between DES and error of refraction 

Studies among medical students in Poland [10], Saudi Arabia [23], and Trinidad and Tobago [14] showed a strong correlation between error of refraction and DES. Similarly, 83.05% of the study cohort had errors of refraction that correlated with DES (p = 0.001). Partially corrected refractive errors, per se, may not directly trigger DES; however, prolonged near work and decreased blinking with the use of VDTs can collectively play a role in the progression or exacerbation of DES [4,10]. In addition, among those with errors of refraction in this study, 43.22% had been using contact lenses, which is a known risk factor for DES. The tear film is separated by the contact lens into the pre- and post-lens tear film, resulting in increased evaporation [4,10,24,25]. Friction between the contact lens and the ocular surface can damage the cornea, thereby aggravating the DES [10,24,25]. Contrary to previous studies [4,10,17,22], no statistically significant correlation (p = 0.091) was found between contact lens wear and DES in this study.

Association between DES and comorbidities, medications, and allergies

Several studies have shown that comorbidities, maintenance medications, and allergies increase the risk or severity of DES [4,10,26]. This study involved youth aged 20-30; hence, only 2.93% had comorbidities, and only 12.28% were using antihypertensive, contraceptive, analgesic, and hypoglycemic medications. Further, 38.14% had allergies that showed a close association with OSDI (p = 0.054), with a mean score of 34 ± 19. Allergy can lead to inflammatory reactions in the eyes. Ocular allergy results in alterations of the mucin, aqueous, and lipid components of the tear film, leading to tear hyperosmolarity [10]. Matrix metalloproteinase (MMP-9), a proteolytic enzyme produced in ocular allergy, leads to corneal epithelial damage that further aggravates DES [10,26].

Association between DES and duration of stay in air-conditioned rooms

Other risk factors that predispose to DES include smoking, use of face masks, and prolonged exposure to air-conditioned rooms [4,5,10]. The majority of the students in this study (83.05%) neither smoked nor had exposure to smokers. Additionally, 48.73% used face masks for more than four hours a day, and 53.81% had exposure to air-conditioned rooms for more than four hours daily. These variables were not statistically correlated with DES in the study, with p-values of 0.732, 0.230, and 0.427, respectively.

Association between DES and duration of use of VDTs

Despite the resumption of regular face-to-face classes, 97.88% of the participants use visual display terminals (VDTs) for more than four hours daily for school work, research, entertainment, and social media. Of these, 82.68% developed mild to severe DES. Continuous use of VDTs reduces the blink rate, which in turn increases tear film evaporation [4]. Decreased and incomplete blinking also reduces Meibomian gland secretion, resulting in a decrease in the lipid component of tears and predisposing individuals to faster evaporation and subsequent dry eye syndrome (DES) [4,10,16]. This study, however, showed no correlation between DES and the use of VDTs (p = 0.124).

Association between DES and psychological stress

Various studies have been published on the psychological impact of the COVID-19 pandemic [9,11-13,21,27-30]. There was an increase in psychological stress and emotional exhaustion, particularly among medical students in their final year [12], and the stress felt by the students correlated with DES [4,12,13,27]. Heightened production of interleukin-1, interleukin-2, interleukin-6, interleukin-8, and tumor necrosis factor alpha (TNF-α) during stress may play a role in ocular surface inflammation leading to DES [10]. Our paper demonstrated a significant direct correlation between the severity of DES and psychological stress (p = 0.006, p = 0.017, and p = 0.0001 using Chi-square, Pearson's, and Spearman's correlations, respectively), further validating previous studies.

The prevalence of psychological stress in this study was 97.88%, similar to studies conducted among medical students in Egypt (85.5%) [28], India (93.3%) [29], and Saudi Arabia (97.1%) [30]. This study also concurs with previous studies that determined younger age and female sex as risk factors associated with higher stress among medical students [12,13,28,30].

Association between DES and sleep duration

There was no correlation between DES and sleep duration in this study (p = 0.196). However, a strong inverse relationship was found between sleeping time and perceived stress (p = -0.141). Students with higher stress levels had shorter sleep times, and vice versa, which is consistent with studies among university students in Croatia [27] and medical students in Saudi Arabia [30], thus confirming that mental stress can result in more severe DES.

Association between psychological stress and duration of use of VDT

A direct correlation between psychological stress and DES was elicited among Indian [4] and Polish [10] medical students. However, this study did not reveal a correlation between the duration of continuous VDT use and DES (p = 0.124). In contrast, there is a highly significant association between the duration of VDT use and psychological stress (p = 0.006). Our research is a cross-sectional study; hence, the interplay between DES, psychological stress, sleeping time, and VDT use was not completely elucidated.

Limitations

Due to the high response rate from the target population and the use of validated questionnaires, including the OSDI and PSS-4, selection bias was reduced. On the other hand, limitations include a focus on only one medical school in the Philippines and a lack of representation of all medical students, thereby reducing the generalizability of this study. Additionally, the study relied solely on questionnaires, without any associated clinical examinations. Testing for tear stability, tear volume, ocular surface assessment, eyelid evaluation, and tear film assays, with the aid of slit lamp biomicroscopy, should be employed as supportive examinations to accurately determine the presence of DES.

## Conclusions

Dry eye syndrome is increasingly recognized as a significant health issue among young individuals, largely due to the increasingly demanding nature of their lifestyles. This study identified a relatively high prevalence of dry eye syndrome at 82.2% and an incidental finding of 97.88% perceived stress among medical students. The female sex, error of refraction, and higher levels of perceived stress are identified as risk factors for developing dry eye syndrome. Our study did not find any significant link between DES and prolonged use of VDTs. Given the significant association between dry eye syndrome and the level of perceived stress, it is crucial to emphasize the importance of early diagnosis and prompt treatment. A longitudinal study involving different medical schools in the country, with the inclusion of clinical tests for DES, is recommended to better elucidate the relationship among the various risk factors. The initial results of this study can serve as a basis for future longitudinal studies, leading to the development of policies that increase awareness, modify risk factors, and implement appropriate preventive measures.
